# Qigong in Cancer Care: Theory, Evidence-Base, and Practice

**DOI:** 10.3390/medicines4010002

**Published:** 2017-01-12

**Authors:** Penelope Klein

**Affiliations:** Physical Therapy Program, D’Youville College, 361 Niagara St, Buffalo, NY 14201, USA; kleinqpj@roadrunner.com

**Keywords:** cancer, Qigong, Tai chi, review

## Abstract

**Background:** The purpose of this discussion is to explore the theory, evidence base, and practice of Qigong for individuals with cancer. Questions addressed are: What is qigong? How does it work? What evidence exists supporting its practice in integrative oncology? What barriers to wide-spread programming access exist? **Methods:** Sources for this discussion include a review of scholarly texts, the Internet, PubMed, field observations, and expert opinion. **Results:** Qigong is a gentle, mind/body exercise integral within Chinese medicine. Theoretical foundations include Chinese medicine energy theory, psychoneuroimmunology, the relaxation response, the meditation effect, and epigenetics. Research supports positive effects on quality of life (QOL), fatigue, immune function and cortisol levels, and cognition for individuals with cancer. There is indirect, scientific evidence suggesting that qigong practice may positively influence cancer prevention and survival. No one Qigong exercise regimen has been established as superior. Effective protocols do have common elements: slow mindful exercise, easy to learn, breath regulation, meditation, emphasis on relaxation, and energy cultivation including mental intent and self-massage. **Conclusions**: Regular practice of Qigong exercise therapy has the potential to improve cancer-related QOL and is indirectly linked to cancer prevention and survival. Wide-spread access to quality Qigong in cancer care programming may be challenged by the availability of existing programming and work force capacity.

## 1. Introduction

Cancer is a human experience, emotional and spiritual as well as physical. Debilitating effects of disease processes and subsequent disease management often have a sustained deleterious effect on quality of life (QOL) in terms of pain, impaired physical mobility and stamina, vitality, sense of well-being, self-efficacy, and social engagement [1]. Integrative oncology addresses the totality of the cancer experience by viewing the individual as a whole, recognizing the mind/body connection, the client-clinician relationship, and client participation, and advocating the integration of the best of both medical and evidence-based complementary interventions [2]. Clinical oncologists routinely recommend exercise in the management of cancer. Its benefits in cancer rehabilitation have been validated [3]. Qigong (pronounced: chee-gong) is a system of mind/body exercise with restorative benefits that potentiates the whole person. When compared to more traditional physical exercise, Qigong practice has been found to have added value in terms of improvements on QOL [4].

## 2. Purpose

The practice of Qigong is not well known in modern Western society [5]. Therefore, the purpose of this discussion is to raise awareness by exploring the applied theory, evidence base in terms of the potential benefits of Qigong within integrative oncology, and practice modalities.

## 3. Methods

Sources for this discussion include a review of scholarly texts, the Internet, and a search of PubMed identifying related research and recent systematic reviews on this topic. The PubMed search used the key words Qigong AND cancer filtered by study type as review. The search was time limited to January 2000 through October 2016, Additional sources include field observations of Qigong in cancer care programming with input from experts in the field and program participants from the US, Canada and Australia [6].

The questions addressed in exploring Qigong in cancer care are: What is Qigong? How does it work? What evidence is there to support its practice in integrative oncology? How is it practiced? What barriers to programming access exist?

## 4. Exploring Qigong in Cancer Care

### 4.1. What Is Qigong?

The broad concept of Qigong may be sub-classified as *spiritual*, *healing*, *medical*, or *martial* Qigong. As translated from the definitive text on Chinese medicine, Qigong refers to all skills of mind-body exercises that integrate breathing adjustment, body adjustment and mind adjustment into one [7]. This is a broad area of therapeutics. Healing and medical Qigong may be passively received as external Qigong or actively practiced as internal Qigong meditative exercises and energy cultivating practices. External Qigong involves the therapeutic transfer of vital energy through a skilled practitioner to the client.

Qigong exercise including Tai chi, performed as Qigong, is the major focus of this discussion. Qigong exercise is an ancient Chinese health care system that integrates physical postures, breathing techniques and focused intention [8]. Qigong exercise is practiced as a meditative (static or dynamic) postural exercise characterized by flowing, rhythmic movements incorporating breath regulation, mindful meditation (intent), and self-massage [9]. Practicing this gentle exercise to optimize the flow of vital energy (Qi) within the body for health is believed to make one more vital, more resistant to disease, and to enhance the clarity of thought and the capacity to learn new things. The art of Tai chi quan (shadow boxing), an exercise more familiar to the Western world, can be practiced as Qigong, However, traditional Tai chi quan movement patterns and forms are often complex and more difficult to master as compared to traditional Qigong exercise due to the martial application of the former. Note that for the purposes of this discussion, the term Qigong exercise includes both traditional and modified internal Qigong exercise as well as traditional and modified Tai chi performed as Qigong. It is of importance to note that internal Qigong practice is much more than a slow, relaxing exercise as it might appear to a naive viewer.

### 4.2. Theoretical Foundations: How Does It Work?

In Chinese medicine, Energy theory is accepted at face value. It originated through processes of intuition, observation, clinical impression, and empirical evidence passed down through millennia from master to student through legacy teaching. The *Yellow Emperor’s Inner Classic*, compiled around the second century B.C.E., with foundational knowledge predating this published work by centuries, is considered the first comprehensive record of Chinese medical theory [10]. It posits the interrelation among humans, their environment, and the cosmos, and the effect of this relationship on human vitality, health and disease prevention and management. Energy theory purports that we possess a vital life energy known as Qi. We are born with a quantity of essential Qi to begin life. The most sustentative origins of this essential Qi are genetic endowment and prenatal nutrition and environment. To sustain life, we replenish our vital energy by accumulation of nutritive Qi through meditation, exercise, the foods and herbs we ingest, the water we drink, the air we breathe, and, even possibly more importantly, our emotional and belief states. The postulate that mindset can influence health, healing and longevity is a root tenet of Chinese medicine.

Qi flow or circulation is mapped into meridians used in acupuncture, energy gates, vessels, and microcosmic and macrocosmic paths within the bioelectric body [11]. Disease is attributed to stagnation or blockages of this vital energy flow.

Scientific validation of energy theory is challenged because Qi, as a physical entity, as yet cannot be directly measured. However, advances in micro-electric, infrared, magnetic, and vibrational biomeasurements hold promise to expand our knowledge of the mechanisms of action. Indirect measures of the biological influence of Qi cultivation do exist [12]. These involve indicators including brain waves, cardiovascular response, physiologic response, biomarkers of immune function, inflammatory mediation, stress, and even structural changes to our brains and our DNA. More modern theoretical explanations for understanding the therapeutics of Qigong include (a) psychoneuroimmunology which is the study of mind/body interactions and their influence on the immune system [13]; (b) the relaxation response effect [14]; (c) the effects of meditation [15]; and epigenetics [16] which is the study of non-genetic coding factors including biological, environmental, emotional, lifestyle, and belief factors that influence genetic expression (switching genes on and off).

### 4.3. Evidence-Based Practice: What Evidence Exists?

If Qigong is to be accepted as a valued therapeutic intervention within our modern health care systems, edicts from legacy teaching and traditions within clinical practice are not sufficient justification for the adoption of innovation. Qigong needs to be subjected to the same scientific investigatory research scrutiny as any other clinically-based health care modalities. Growing acceptance of the field of integrative oncology, combined with a dedicated research agenda and funding availability, have resulted in exponential growth of published research investigating the theory and therapeutic value of Qigong in recent years. A 2015 extensive bibliographic review of therapeutic Tai chi (performed as internal Qigong) by Yang et al. examined over 500 studies [17]. This research evidence crossed most major clinical areas including cancer. Nearly 95% of the studies reported positive effects in one or more primary health outcomes studied with no evidence of serious adverse effects.

The body of research addressing Qigong in cancer care is much more limited. Past challenges included few high quality studies. However, the existence of high quality research evidence in this area is growing. The growth and strength of new evidence is demonstrated in the chronology of conclusions of recent systematic reviews assessing the effects of Qigong in cancer care. A search of research evidence through 2010 by Chan et al. [18] concluded that due to a paucity of studies and a high risk of bias and methodological problems in the majority of topic-related studies, it was still too early to draw conclusive statements. Further, these researchers concluded that vigorously designed, large-scale, randomized clinical trials (RCTs) with validated outcome measures were needed. A more recent systematic review by Zeng et al. (2014) concluded that emerging evidence did demonstrate that Qigong/Tai chi had positive effects on the QOL, fatigue, immune function, and cortisol levels of cancer patients [19]. These researchers stated that results should be viewed cautiously due to the limited study quality. In 2016, Klein et al. [20] raised the bar on the quality of evidence being analyzed by a systematic review limiting study inclusions to RCTs with a minimum of 15 subjects per group by protocol. Eleven qualifying studies [21,22,23,24,25,26,27,28,29,30,31] examining a total of 831 subjects, using nine different Qigong protocols, were included in this most recent systematic review. These researchers validated the initial conclusions of Zeng et al., stating that the practice of Qigong positively influenced quality of life (QOL), fatigue, immune function and cortisol levels, and cognition in individuals with cancer [32]. Ongoing research promises to continue to inform this topic.

While testimonials of individuals attributing the personal practice of Qigong with curing their cancers abound on the Internet, as of yet, no strong body of research evidence validating these claims exists. There is, however, indirect evidence that suggests the potential benefits of Qigong practice with regard to cancer prevention. The Shanghai Study, a large, prospective, longitudinal study being conducted in China, has identified exercise as a protective lifestyle factor influencing reduced cancer mortality rates [33]. The regular practice of Qigong exercise is known to boost the immune system and mediate the inflammatory response [24,34], two factors linked to cancer prognosis. Further, there is preliminary evidence that Qigong practice can influence the repair of telemeres [35]. Telemeres are chromosome ends, a specialized structure involved in the replication and stability of DNA molecules. Telemere damage has been associated with a poor prognosis for cancer survival [36]. This collective body of emerging evidence provides a rationale for future, objective, more rigorously controlled evaluations of the benefits of Qigong exercise practice with regards to cancer prevention and management. Foundational to modern scientific study validation, there are more than a thousand years of empirical evidence within the practice of Chinese medicine.

### 4.4. How Is Qigong Practiced?

#### 4.4.1. Exercise Forms

There may be as many styles and forms of Qigong exercise as there are Qigong masters. These include many form variations of mind-body exercises and meditations. Included among the older forms and styles are Daoyin [37], Baduanjin (Eight pieces of Brocade) [38], Zhan Zhuang (standing pole) [39], the Five Animals [40], Yijinjing [41], and Dragon Tiger Medical Qigong [42]. Descriptions of Qigong exercises that form the basis for many of the modified modern Qigong forms can be found in a 1984 English translation of Qigong exercises compiled by a committee of Chinese Qigong experts including Master Gu Daifeng, Dr. Cao Xizhen, Dr. Wang Ziping, and Master Guo Lin [43].

A variety of derivative Qigong forms have been employed as research protocols and found to be effective in supportive cancer care [21]. These include Medical Qigong, Guolin Qigong, Tai Chi Chih, Qigong/Tai Chi Easy™, Kuala Lumpur Qigong, 8-Form Tai Chi, and the standardized International 24-movement Tai Chi form.

Guolin Qigong, a form made popular in China as specific for cancer care, is recognized by its stylized walk accentuated by a coordinated arm swing, resultant trunk rotation, and breath regulation. This walking exercise is complemented with basic, intermediate and advanced forms performed as gentle exercise. Acupressure point stimulation is self-applied as body tapping or drumming or through external Qigong. The comprehensive practice of Guolin Qigong also includes social support and education in nutrition and stress management [44].

#### 4.4.2. Demystifying Practice

Regardless of style, dynamic Qigong exercise can be experienced in three progressive levels deepening the mind/body connection and benefit potential with skill advancement. The first level of practice is physical. At its base level, practitioners learn to master a series of postural corrective exercises as well as techniques for self-massage. Benefits of physical practice include brain activation associated with new learning, balance, strength, neuromotor coordination, postural correctives and visceral stimulation. On the second level, breathing is integrated within movement patterns or static postures inducing mental calm and a relaxation effect. Again, practice at this level does not provide the full benefit of traditional Qigong practice. It does provide the additional benefit of dynamic progressive relaxation. At the third level, meditative intent (Yi) cultivates energy. *Yi dao, Qi dao*. translates to: *Where the mind goes, the energy flows*. Mindful meditation involves clearing the mind of all distractions (letting go) and allowing or directing the accumulation, circulation, and storage of energy from our environment into and through our body electric. The following proposes an expanded explanation for mechanisms of action at each progressive level of dynamic exercise practice: physical, physical plus breath regulation to induce relaxation, and mindful or meditative Qigong.

The physical component of dynamic Qigong exercise patterns can be deconstructed as multi-dimensional. On a biomechanical level, many of the exercises serve as postural correctives increasing flexibility and restoring muscular and soft tissue structural balance as well as providing a gentle, regenerative, mechanical stimulation of articular surfaces. General benefits of exercise including benefits to cardiovascular and immune function are also realized. Most basic Qigong exercises and simplified Tai chi forms can be adapted to activity tolerance for performance in standing, sitting, or lying postures.

Dynamic movement patterns of traditional Qigong combine biomechanical effects and self-massage. Self-massage has the potential to stimulate acupuncture points along meridians, open energy gates, and provide visceral massage. Self-massage may be applied directly as gentle circular massage, as body drumming or tapping, and as acupressure (e.g., face or foot massage) [45], or indirectly through movements such as slow, reversing trunk rotations that induce gentle internal visceral and lymphatic massage. High frequency, low amplitude vibrations transmitted to vital organs through sustained vocalizations, known as healing sounds [46], can also be considered a form of self-massage. Other massage techniques include auric massage where the hand strokes the energy field lying adjacent to the limits of the physical body, very much like self-applied Reiki or Therapeutic Touch.

It is widely accepted that stress threatens health. Training in relaxation is commonly used as a stress management strategy. Breath regulation during Qigong exercise induces a calming relaxation response. The relaxation response stimulates the parasympathetic nervous system [14]. Activation of the vagus nerve slows and deepens respiration, increasing oxygen levels and dilating arteries, thereby lowering cardiovascular resistance and subsequently lowering blood pressure. Additional beneficial effects include improved blood flow to the brain, altering of brain waves associated with a sense of well-being, boosting the immune system, and regulation of blood sugar levels.

Meditation is an integral and root component of traditional Qigong exercise practice [47]. It is known to have a wide range of health benefits [48]. Qigong experts use a variety of modes of meditation to advance practice and cultivate Qi. Qigong instructors traditionally emphasize mindful exercise, encouraging the development of an inner awareness during exercise.

Mindfulness is a term often associated with Qigong exercise. Through mindfulness, one develops a heightened sense of inner awareness. One is instructed to focus on breathing and all sensations associated with the exercise being performed, be it quiet posturing or active movement. Mindfulness allows one to stay in the moment and to avoid distractions of thought or external stimuli. In addition to stress management and inducing the relaxation effect, mindful meditation has been proven to change brain structure. In an eight-week study of novice practitioners, mindful meditation, learned as part of a stress reduction program, was associated with changes in the gray matter concentration in brain regions involved in learning and memory processes, emotion regulation, self-referential processing, and perspective taking [49]. Two other modes of meditation commonly used in Qigong practice include (a) emphasis on emptying the mind fully to allow the Qi to flow naturally; and (b) direction to focus on inward visualization or intentionally directing the flow of the Qi. Each strategy has utility and Qigong instructors often employ multiple strategies to serve the intended purposes (see Table 1 for comparative examples of modes of meditation instruction). In mental application, intent, meditation, heightened awareness, and a healing mind set, essential to Qigong practice, are collectively considered the true “golden treasure” of Qigong.

Initially, meditative Qigong may best be learned and practiced in static postures: lying, sitting, or standing. In addition to instruction in mindful dynamic exercise, Qigong practice often includes quiet meditation which may be practiced while lying, sitting or standing. Taiji Five-Element Qigong (TFQ), a formless Qigong system developed by Master Binhui He, employs Daoist meditation and visualization to cleanse, energize, boost the immune system and purify the body. In 1996, a group of 13 scientists and medical professionals, gathered in Guangzhou City, (Guandong, China), concluded that “the achievement of anti-cancer therapy by Chinese Taiji Five-Element Qigong is significant for humans to overcome cancer, and should be promoted to the public” [58].

It is only when free, flowing exercise movements are automatic, after many repetitions of practice, that they can be performed in a meditative state. With the complex movement patterns of traditional Tai chi, this may take many years of practice, if ever achieved. Employing simplified movements and forms, characteristic of Qigong and modified Tai chi, makes the goal of reaching a meditative state during dynamic exercise more attainable. Because achieving a meditative state is conceivably more important to the individual with cancer than mastering complex movement patterns and forms, Qigong or modified easy-to-learn Tai chi with a limited number of dynamic postures is considered most appropriate for use with this clinical population.

## 5. Discussion

Sometimes what is old is new. While ancient in origin, the practice of acupuncture was only introduced to the West in the 1970s. Today, it is well known. Similarly, Qigong is ancient, but can be considered as innovative within Western health care. The adoption of health care innovation follows a familiar pattern: empirical interest, the establishment of efficacy, theory validation, service demand and delivery logistics.

Given the strength of evidence of effect and advancement on theory validation, we are rapidly moving into service demand and delivery logistics. There are an estimated 15 million cancer survivors in the United States alone [59], and another estimated 15 million newly-diagnosed cases annually worldwide [60]. This incidence and prevalence combined with the belief that advances in cancer treatment will increase survivorship, such that many will experience cancer as a chronic illness, establishes the potential of a high service need. The challenges to meeting the adoption of Qigong as a therapeutic intervention within Western integrative oncology are many. Among these challenges are limited awareness of the benefits of Qigong exercise among the public and health care communities, as well as limited programming availability, lack of consensus on programming structure, and a need to build work force capacity.

Public and professional awareness can be raised through education via professional presentation to peers and to the public through media presentations such as the YouTube postings by Dr. Yang that include an informative video series featuring participants of the program at the Memorial Sloan Kettering Cancer Center [61].

Dedicated programming for Qigong in cancer care availability to meet a potential future demand is lacking. A few major cancer centers such as the MD Anderson Cancer Center, Houston TX, the Dana-Farber Cancer Institute, Boston, MA, and the Memorial Sloan Kettering Cancer Center currently list Qigong programming as an option among a schedule of support services. Dr. K. Chen, University of Maryland, School of Medicine, offers a seven-day, self-healing retreat utilizing a Qigong system designed specifically for managing cancer [62]. The retreat includes instruction in meditative exercise as well as stress management strategies related to changing how one thinks, perceives, and responds to life experiences. Wellspring Niagara, an independently funded cancer support service organization affiliated with regional centers across Canada, is an example of community-based program [63]. It has been offering free Qigong group classes to clients served for over 15 years. However, these resources are the exception rather than the rule.

Given that limited availability of qualified Qigong instructors challenges the capacity to meet an expanding service need, the question then arises: how do we plan to meet this need? There is no single, universally recognized Qigong accrediting body to assure Qigong instructor competence. The National Qigong Association (NQA) does independently certify Qigong instructors who meet a standard of a minimum of 200 h of qualified instruction. The NQA numbers approximately 450 members from six countries. This is a small number considering the potential need. Free-standing instructor training programs do exist for many Qigong styles. These training courses can be as short as a weekend introduction to a specific form to more comprehensive study over two to three years that generally includes energy medicine theory and therapeutics. One suggestion as a mechanism to expand the Qigong work force is to consider cross-training exercise and rehabilitation health care professionals. For example, schools of Physical Therapy might enlist Qigong experts to add instruction in Qigong as a therapeutic exercise to entry-level and continuing professional education curricula.

Beyond the actual number of instructors is a question of competence. Existing, entry-level Qigong training may be sufficient for serving healthy or generally healthy aging populations, but training may need to be augmented to meet the specific needs of the clinical population of individuals with cancer. Concurrent with future research of populations addressing prevention and long-term clinical studies assessing cancer-related survival benefit, applied research to establish consensus guidelines for Qigong in cancer care programming structure would serve society. Such guidelines could assist to assure future programming quality and to guide Qigong instructor training to meet the anticipated future service need in supportive cancer care.

## 6. Conclusions

While many research questions remain unanswered, there is sufficient evidence supporting the belief that Qigong exercise has a complementary role in supportive cancer care. There are established benefits validating the potential of Qigong practice to improve cancer-related QOL with no known serious side effects. Qigong practice has been linked to prevention and improved cancer-related mortality rates. Challenges to wide-spread Qigong in cancer care programming adoption include (a) lack of awareness and general knowledge among the public, health care providers and programming administrators regarding the effectiveness of Qigong in cancer care; (b) limited availability of existing programming to meet potential need; and (c) limitations in work force capacity to meet the anticipated expanding demand for dedicated programming.

## Figures and Tables

**Table 1:** Examples of Qigong mediation modes: empty mind vs. visualizing and directing Qi flow vs. heightened inner awareness.

Mode	Concept	Instruction	Source
Empty mind	Inward Contemplation	“… a sense of lightness, peace, calmness, and tranquility.”	Master Yang Yang, PhD [50]
	Quiescence	“Dissolving in Qi, I merge with the boundless universal field of being.”	Roger Jahnke, OMD [51]
	Tranquility	“Allow yourself to become empty. Abide in stillness”	Lao Tzu [52]
Visualizing and directing of Qi flow	Entering tranquility	“A quiet mind can sense imbalance more easily and is better able to direct the flow of Qi.”	Kenneth S. Cohen [53]
	Mind and Qi Harmony	“… You can harmonize your body, breathing, mind, Qi, and spirit to a deep meditative stage.”	Master Yang, Jwing-Ming, PhD [54]
	Directing Qi	Trace the Yin Yang Channels, Direct Qi to the organs, and Direct Qi in the Micro-cosmic orbit.	Roger Jahnke, OMD [55]
Mindful Meditation: Heightened inner awareness	Focused Meditation	“Awareness of moment-to-moment sensations allows you to train and hold your mental focus, …”	Peter Wayne, PhD [56]
	Three natures return to one	“…think, look, listen to your [middle] dantien …”	Master Yang Yang, PhD [57]
